# Impact of Tissue Freezing on the Functional and Biomechanical Characteristics of Porcine Aortic Roots

**DOI:** 10.1007/s13239-026-00833-1

**Published:** 2026-05-20

**Authors:** Tanita Drejer Jeppesen, Johannes Høgfeldt Jedrzejczyk, Anne Lund Vingborg, Alberte Damgaard Schmidt Hansen, Søren Nielsen Skov, Leila Louise Benhassen, J. Michael Hasenkam, Peter Johansen

**Affiliations:** 1 Department of Clinical Medicine, Aarhus University, Aarhus, Denmark; 2 Department of Cardiothoracic and Vascular Surgery, Aarhus University Hospital, Palle Juul-Jensens Boulevard 99, 8200 Aarhus N, Denmark; 3 Department of Electrical and Computer Engineering, Aarhus University, Aarhus, Denmark

**Keywords:** Cardiovascular experimental research, Aortic root, Aortic valve, Tissue preservation, Biomechanics, In vitro model

## Abstract

**Purpose:**

Freeze-preservation of porcine aortic tissue is often necessary for experimental planning and standardization, but the impact of freezing on aortic root properties has not been systematically investigated. This study evaluated the effects of freezing and thawing on the biomechanical, fluid dynamic, and ultrasonographic characteristics of porcine aortic roots.

**Methods:**

A total of 32 porcine hearts were obtained from a local abattoir. Specimens were randomized to a fresh group (immediate testing) or a thawed group (storage at − 20 °C for 30 days followed by thawing in saline). Uniaxial tensile testing was performed on samples from the sinotubular junction (STJ) and non-coronary cusp (NCC) (n = 10 per group). Functional testing was conducted in a pulsatile flow loop (n = 6 per group), with mean aortic pressure gradient, forward and retrograde flow measured and the regurgitant fraction calculated. Ultrasonography was used to assess annular diameters and coaptation lengths.

**Results:**

Tensile testing revealed no statistically significant differences in E-module, maximum stress, maximum load, or stiffness between fresh and thawed specimens at either the STJ or NCC. Mean aortic gradient, retrograde flow and regurgitant fraction did not differ. Ultrasonography showed no statistically significant changes in annular geometry or leaflet coaptation length after freezing and thawing.

**Conclusion:**

Freezing and thawing did not result in measurable differences in the biomechanical, fluid dynamic, or geometrical properties of porcine aortic roots under the tested protocol and within the sensitivity of our measurements. These findings support the use of thawed porcine aortic roots in experimental cardiovascular research.

## Introduction

Pulsatile left-heart flow-loop models are widely used to investigate aortic root biomechanics and fluid dynamics [1, 2]. These systems mimic cardiac dynamics, allowing for a detailed assessment under both physiological and pathological conditions. They are commonly used to study aortic root function, evaluate valve performance, and test cardiovascular devices. Porcine hearts are frequently selected for these experimental setups owing to their close anatomical and biomechanical resemblance to human hearts [3, 4].

Fresh or short-term refrigerated porcine specimens are often used, but maintaining standardized experimental conditions can be challenging. Variability arises from differences in post-harvest handling times, storage conditions, and specimen availability. Refrigeration at 4 °C has been reported to reduce tissue integrity, resulting in lower elasticity, sub-failure properties, and ultimate strength relative to fresh or frozen specimens [5]. On the other hand, appropriately frozen porcine aortic tissue often exhibits biomechanical properties comparable to those of fresh samples [5]. In descending porcine aortas, studies report no significant changes in biaxial mechanical performance after long-term freezing [6] and no detectable alterations in geometry on ultrasound [7].

However, the effects of tissue freezing on vascular biomechanics remain poorly understood, and conflicting results have been reported in the literature. While several studies suggest that freezing preserves mechanical integrity of arterial tissue, others have demonstrated alterations in stiffness, strength, or microstructural organisation following cold storage or freeze–thaw cycles. Venkatasubramanian et al. reported freeze–thaw–induced changes in arterial mechanics linked to collagen and smooth muscle behavior [8], whereas Chow, and Zhang and Hemmasizadeh et al. observed storage-dependent alterations in mechanical and biochemical properties using different testing modalities and tissue types [9, 10]. These discrepancies likely reflect differences in tissue type, anatomical region, storage temperature, duration, freezing protocol, and mechanical endpoints assessed.

Importantly, most prior studies have focused on straight arterial segments or isolated wall specimens, whereas the aortic root represents a structurally and functionally complex unit integrating annular support, leaflet motion, and sinotubular dynamics. Whether findings from simpler vascular geometries can be extrapolated to the aortic root therefore remains uncertain, underscoring the need for targeted evaluation using integrated functional and biomechanical assessments.

Whether comparable findings apply to the aortic root and valve apparatus remains uncertain, despite their central role in modelling physiological biomechanics and device performance. Addressing this knowledge gap is essential to ensure that in vitro models replicate clinically relevant tissue behavior and provide valid platforms for cardiovascular research.

We therefore tested the null hypothesis that thawed and fresh porcine aortic roots do not differ in (1) ultimate tensile properties and (2) in vitro flow-loop performance, assessed by ultrasound-derived geometric measures and quantified regurgitant fraction.

## Methods

### Study Design and Materials

The porcine species was chosen for this in vitro study due to its similarity with human heart physiology and anatomy [4]. A total of 32 porcine hearts were harvested from a local abattoir. The aortic roots were inspected for macroscopical pathological abnormalities, and the intraluminal diameters of the annulus and sinotubular junction (STJ) were measured using valve sizers. The coronary arteries were then ligated with 3-0 Novosyn ligatures (B. Braun, Melsungen, Germany). Following dissection as previously described [1], specimens were randomized into a fresh or a thawed group. In the flow-loop experiments, each aortic root served as its own control, being evaluated first in the fresh state and subsequently after freezing and thawing.

In contrast, uniaxial tensile tests were performed on separate fresh and thawed samples, as the required tissue segments from the STJ and non-coronary cusp (NCC) could not be reused once they were excised for mechanical testing. In the fresh group, ten roots underwent immediate uniaxial tensile testing, and six were evaluated in the flow loop. In the thawed group, specimens were stored at − 20 °C for 30 days. Following dissection, aortic roots were placed in sealed containers and frozen directly at − 20 °C without immersion in a liquid medium. No controlled cooling or pre-cooling protocol was applied prior to freezing. The frozen aortic roots were thawed by full immersion in a 22 °C, 4.5 L saline solution for 5 minutes before undergoing uniaxial tensile testing and flow-loop analysis.

### Uniaxial Mechanical Tensile Testing

Tissue samples from the NCC and the aortic wall at the STJ (n = 10 per group) underwent uniaxial mechanical tensile testing. All tissue samples were excised and tested in the circumferential direction for both regions. All tissue samples were cut into hourglass-shaped specimens with a central gauge area of 4 x 3.5 mm using a dedicated puncher to ensure uniformity and reproducibility of the samples [11]. The sample width and length were measured with calipers, and thickness was determined using a gauge applying a constant pressure of 18 kPa (Model 7301, Mitutoyo Corporation, Kawasaki, Japan).

Prior to tensile testing, the fresh and thawed tissue specimens were immersed in a saline solution (0.9% NaCl, Fresenius Kabi, Fresenius SE & Co. KGaA, Bad Homburg vor der Höhe, Germany) for 10-15 minutes to ensure hydration. During uniaxial tensile testing, tissue specimens were continuously hydrated by periodic saline spraying at approximately two-minute intervals. Specimens were immersed in a saline bath immediately before and after testing to minimize dehydration during mechanical loading.

Mechanical testing was performed using a Bose ElectroForce 3200 system (Bose Corporation, ElectroForce Systems Group, Minnesota, USA) equipped with a 225 N load cell (± 0.002 N). Specimens were mounted between two custom-made gripping clamps (G341-10-32 Needlenose Screw Vice Grip Rigid Mount, TestResources Inc., USA). The inter-clamp distance was increased at a constant displacement rate of 0.5 mm/s, with data acquired at 10 Hz. Each specimen was subjected to uniaxial tensile testing until failure (typically at a displacement of approx. 10 mm), while both force and displacement were continuously recorded. Displacement was derived from crosshead movement, while force was measured by the load cell. Stress-strain parameters were derived from the recorded force and crosshead displacement signals. Engineering stress was calculated as force divided by the initial cross-sectional area, and engineering strain as displacement relative to the initial gauge length.

Because the tissue response was nonlinear, the elastic modulus was reported as a region-specific modulus (descriptive metric) rather than a linear-elastic material constant. For each specimen, an approximately linear segment was identified in the high-strain region prior to failure, and the tangent modulus was calculated as the slope between the endpoints of this segment.

1 $$E=\frac{{\sigma}_{2}-{\sigma}_{1}}{{\varepsilon}_{2}-{\varepsilon}_{1}}$$

**Fig. 1:**
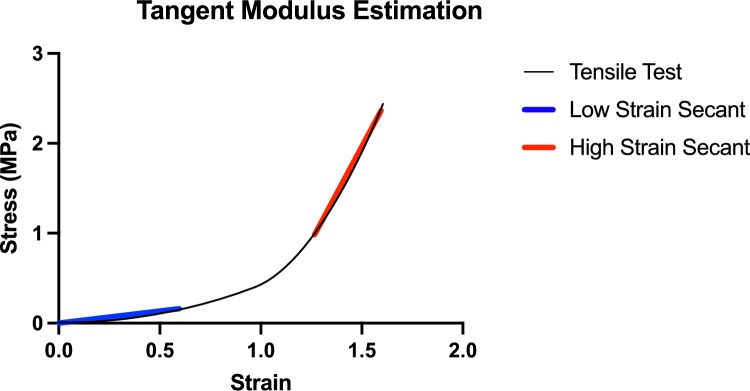
Representative engineering stress-strain curve illustrating the nonlinear response and the basis for modulus extraction. Secant lines illustrate the high-range tangent modulus (high-strain region prior to failure) and, for STJ specimens, the low-strain tangent modulus, prior to the transition to the steeper high-strain region

For the STJ wall specimens, an additional low-strain tangent modulus was calculated analogously from an approximately linear segment prior to the transition to the steeper high-strain region. As the location of these approximately linear regions varied between specimens, endpoint selection was performed individually for each curve using the same criterion across groups. The nonlinear tissue response under uniaxial loading and the basis for parameter extraction are illustrated by a representative engineering stress-strain curve in Fig. 1. Stiffness was calculated in a similar way as the slope of the force–displacement curve over the corresponding high-load region.

#### In Vitro Setup

**Fig. 2:**
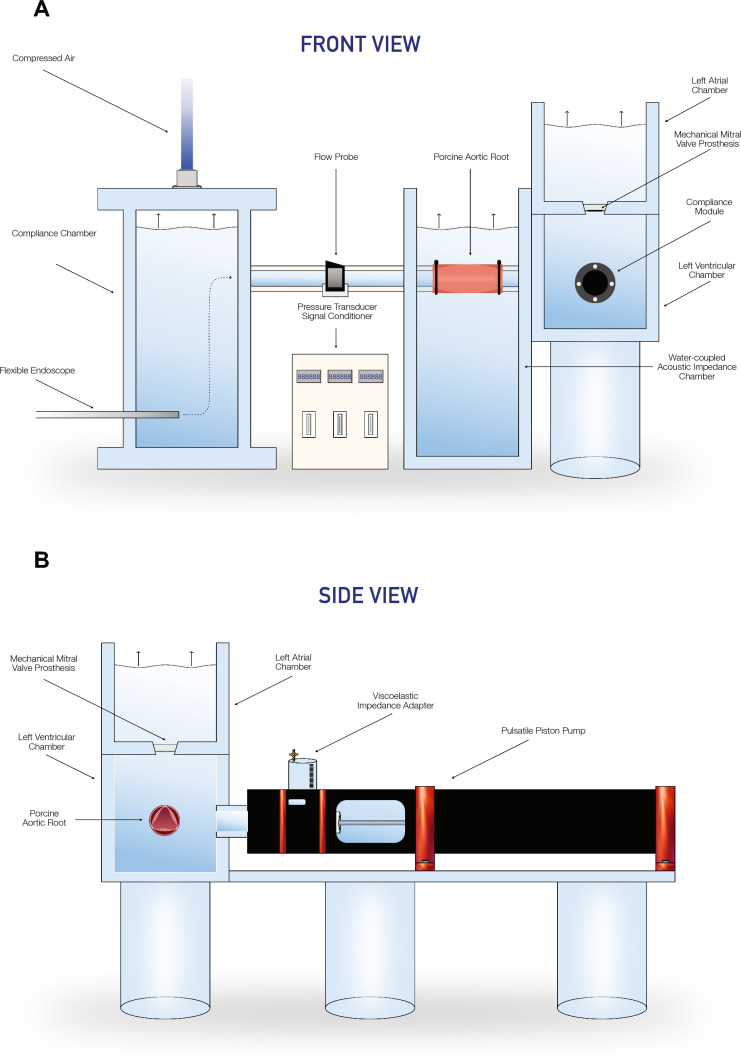
Pulsatile left-heart flow-loop model for in vitro testing of porcine aortic roots, shown in **A** front view and **B** side view

The pulsatile left heart model illustrated in Fig. 2 has been previously described in detail [1, 2, 12]. In summary, the system consisted of an atrial chamber and a ventricular chamber, separated by a mechanical mitral valve prosthesis. The pulsatile flow-loop was operated using demineralized water as the working fluid for all experiments. The ventricular chamber was actuated by a digitally controlled piston pump (SuperPump AR Series, ViVitro Labs, Victoria, Canada), which produced pulsatile flow to simulate left ventricular ejection in the aortic root. The aortic root was positioned within the interchangeable aortic section of the model. A compliance chamber was attached to represent arterial elasticity, and systemic vascular resistance was regulated using an adjustable Hoffmann clamp. Aortic outflow and peripheral venous return were measured with an ultrasonic transit-time flowmeter (PXL11, PXL25, TS410, Transonic Systems Inc., Ithaca, NY, USA). Pressures in the left ventricular and aortic chambers were recorded using fluid-filled tubes connected to Edwards Truwave (Edwards Lifesciences, Irvine, CA, USA). The pressure transducers were connected to a custom-made amplifier unit. Fluid dynamic signals were sampled at 1000 Hz (NI USB-6259, National Instruments, TX, USA), and recordings were managed with custom-written LabVIEW software (version 14.0, National Instruments). The aortic root was visually examined for signs of pathology such as leaflet rupture, calcification, or incomplete closure, using a flexible endoscope (aScope^TM^ 4 Broncho Large 5.8/2.8, Ambu A/S, Ballerup, Denmark).

Ultrasonographic imaging was performed immediately after completion of the fluid-dynamic measurements, with the aortic root maintained in the pulsatile flow-loop setup under identical operating settings.

### Operation Conditions

To replicate physiological left ventricular conditions, the pulsatile flow-loop was set to target aortic pressures of approximately 120/70 mmHg at a heart rate of 70 beats/min, with a target forward flow of 5 L/min. Actual forward flow was 4.84 ± 0.04 L/min in thawed specimens and 4.64 ± 0.07 L/min in fresh specimens. Forward flow represents an operator-controlled variable that is inherently sensitive to minor adjustments in system resistance and compliance. Forward flow was adjusted during setup by minor modifications of stroke volume, system resistance, and compliance to achieve target physiological pressure conditions. As forward flow represents an operator-controlled setpoint rather than an outcome measure, small differences between conditions reflect setup adjustments and do not influence the interpretation or comparison of pressure gradients or regurgitant fraction. Therefore, it does not affect other fluid dynamic measurements and has no physiological significance in the setup. Apart from forward flow, all other fluid dynamic parameters were comparable between fresh and thawed specimens.

### Data Acquisition and Data Analysis

Tensile testing data were collected using the manufacturer’s dedicated hardware and software. Force and displacement signals were obtained directly from a crosshead load cell, with specimen thickness and a fixed gauge length of 4 mm incorporated into the calculations. From these measurements, the maximum load before rupture and the uniaxial stiffness of each sample were determined.

The recorded signals from the flow loop were processed offline using custom virtual instrumentation software (LabVIEW version 14.0, National Instruments). Once the target fluid dynamic conditions were established, fifteen consecutive heart cycles were recorded, and the ensemble average of the pressure and flow recordings was calculated. Systolic and diastolic pressures were defined as the peak and nadir values of the aortic pressure trace. Mean aortic pressure gradient was computed as the time-averaged distance between ventricular and aortic pressures over the systolic ejection phase, defined as the interval from 10% of peak aortic flow before the peak to 10% after the peak. Beat-wise gradients were calculated for each cardiac cycle and then averaged across cycles.

Ultrasonographic imaging of leaflet and annular dynamics and dimensions was performed using a 2D-linear probe (GE Vivid S5, GE Vingmed Ultrasound AS, Horten, Norway). A short-axis view of the aortic root was acquired at the annular level, and a long-axis view was obtained through the right-left coronary interleaflet triangle to include the entire root. The image data was stored for offline analysis using Horos v4.0.0 (Horos Project, Annapolis, MD, USA). Systole and diastole were defined as the phases corresponding to the maximum and minimum annular diameters, respectively. The acquired ultrasonographic parameters were annular internal base diameter at systole and diastole, and coaptation length.

### Statistical Analysis

Continuous variables are summarised in tables as mean ± SD (and median [range] where relevant), whereas boxplots are used in figures to visualise distributions using the median and interquartile range.

All statistical analyses were performed using Stata 19 (StataCorp, College Station, TX, USA). Data were analyzed according to study design: (i) paired analyses were applied when each aortic root was examined in both the fresh and thawed state, and (ii) independent group analyses were applied when fresh and thawed roots originated from separate specimens.

For paired data, each specimen served as its own control. The distribution of within-pair differences was examined using the Shapiro-Wilk test and visual inspection of histograms and Q-Q plots. If the normality assumption was satisfied, paired t-tests were used to compare means. Results are reported as mean ± standard deviation (SD) for each state and as mean paired differences with 95% confidence intervals (CI).

For independent data, descriptive statistics were calculated as mean ± SD, median, and range. The distribution of each variable within groups was assessed using the Shapiro-Wilk test and Q-Q plots. Welch’s two-sample *t*-test was used as the primary method for comparing means between independent groups, given its robustness to unequal variances. When the normality assumption was violated or considered questionable based on Shapiro-Wilk testing and Q-Q plot inspection, the Mann-Whitney *U* test was additionally performed as a non-parametric sensitivity analysis to assess the robustness of the findings.

Group differences are presented as mean differences with 95% CI. A two-sided p-value < 0.05 was considered statistically significant for all analyses.

Sample size for the flow-loop experiments was determined a priori based on previous studies using comparable pulsatile left-heart flow-loop configurations and outcome measures. Power estimation was performed assuming a two-sided significance level of α = 0.05 and a statistical power of 80% ($\beta =0.20; {Z}_{1-\alpha /2}=1.96;{Z}_{1-\beta }=0.84$).

The study was designed to detect a 15% difference (Δ) in the primary functional/ultrasonographic endpoints, considered the smallest effect size of practical and physiological relevance. Based on prior experimental experience, the standard deviation of within-specimen differences was conservatively estimated to be ${\sigma}_{d}\le 10\%$ of the mean. Using the standard sample size formula for paired comparison of two means.

2 $$n=\frac{2({Z}_{1-\alpha /2}+{Z}_{1-\beta }{)}^{2}{{\sigma}_{d}}^{2}}{{\Delta }^{2}}$$

The required sample size was n≈3.9, corresponding to a minimum of four specimens. To align with prior studies and account for potential attrition or measurement failure, six aortic roots were included in the paired flow-loop protocol.

Although no statistically significant differences were observed, the paired flow-loop and ultrasonographic analyses were performed in a limited sample size. While the study was powered to detect moderate differences (~ 15%) between fresh and thawed conditions, smaller effects cannot be excluded. Consequently, non-significant findings should be interpreted in conjunction with effect estimates and confidence intervals rather than as evidence of strict equivalence, and the potential for type II error should be acknowledged.

## Results

### Uniaxial Mechanical Tensile Testing

#### STJ

**Fig. 3:**
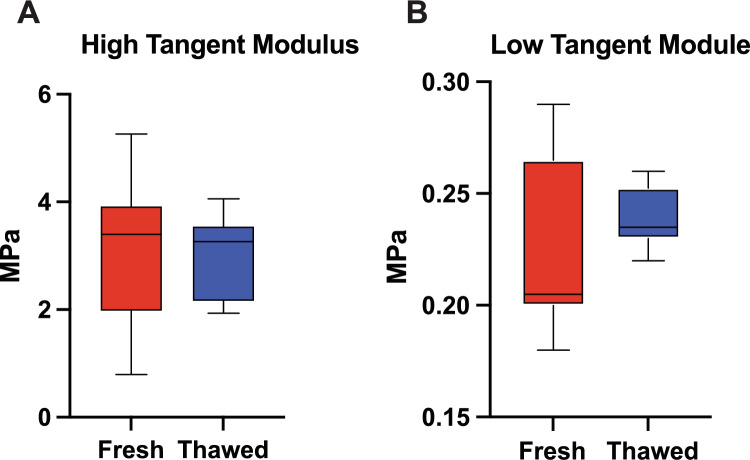
Boxplots illustrating uniaxial tensile testing of the aortic wall at the sinotubular junction in fresh and thawed porcine aortic roots. **A** High-range elastic modulus. **B** Low-range elastic modulus. Boxplots show the median (centre line), interquartile range (box), and whiskers extending to 1.5 × IQR

**Fig. 4:**
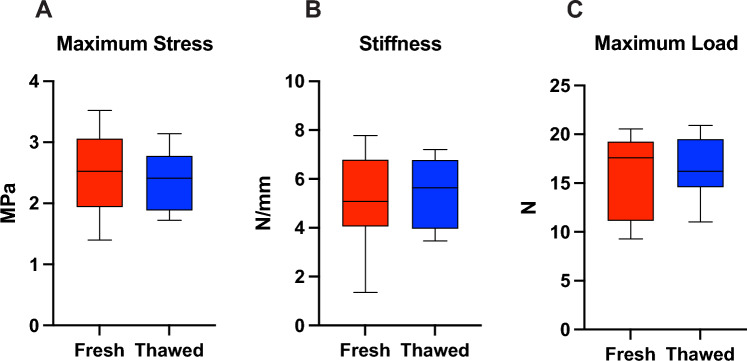
Boxplots illustrating uniaxial tensile testing of the aortic wall at the sinotubular junction in fresh and thawed porcine aortic roots. **A** Maximum stress. **B** Stiffness. **C** Maximum load. No statistically significant differences were observed between fresh and thawed specimens for any parameter (maximum stress: p = 0.64; stiffness: p = 0.73; maximum load: p = 0.67). Boxplots show the median (centre line), interquartile range (box), and whiskers extending to 1.5 × IQR. p-values are from Welch’s *t*-test; Mann–Whitney *U* was performed as a non-parametric sensitivity analysis

Uniaxial tensile test results for the STJ are presented in Table 1 and depicted in Figs. 3 and 4. The high-range elastic modulus was 3.1 ± 1.3 MPa in fresh specimens and 3.0 ± 0.8 MPa in thawed specimens, with no difference detected (mean difference 0.1, 95% CI − 0.9 to 1.1, p = 0.88; Mann-Whitney p = 0.65). The low-range elastic modulus was 0.22 ± 0.04 MPa in fresh specimens and 0.24 ± 0.01 MPa in thawed specimens. As the distribution was skewed, values were analysed after log-transformation, with no statistically significant difference detected between groups (log-transformed mean difference − 0.08, 95% CI − 0.20 to 0.03, p = 0.136).

**Table 1:** Tensile test of the STJ, comparing fresh and thawed tissue

Parameter	Fresh (n = 10) mean ± SD	Median (range)	Thawed (n = 10) mean ± SD	Median (range)	Mean difference (95% CI)	Welch’s *t*-test	Mann-Whitney
Tangent modulus, low (MPa)	0.22 ± 0.04	0.21 (0.18–0.29)	0.24 ± 0.01	0.24 (0.22–0.26)	− 0.08 (− 0.20 to 0.03)	p = 0.136	p = 0.131
Tangent modulus, high (MPa)	3.1 ± 1.3	3.4 (0.8–5.3)	3.0 ± 0.8	3.3 (1.9–4.1)	0.1 (− 0.9 to 1.1)	p = 0.88	p = 0.65
Maximum stress (MPa)	2.5 ± 0.7	2.5 (1.4–3.5)	2.4 ± 0.5	2.4 (1.7–3.1)	0.1 (− 0.5 to 0.7)	p = 0.64	p = 0.65
Stiffness (N/mm)	5.2 ± 1.9	5.1 (1.4–7.8)	5.4 ± 1.4	5.6 (3.5–7.2)	− 0.3 (− 1.8 to 1.3)	p = 0.73	p = 0.82
Maximum load (N)	16.0 ± 4.2	17.6 (9.3–20.6)	16.7 ± 3.2	16.2 (11.0–20.9)	− 0.7 (− 4.2 to 2.8)	p = 0.67	p = 0.82

Biomechanical parameters of fresh and thawed porcine aortic roots. Values are presented as mean ± standard deviation (SD), median (range), mean differences with 95% confidence intervals (CI), and corresponding p-values from Welch’s two-sample *t*-test and Mann-Whitney *U* test. No statistically significant differences were observed between groups

Maximum stress was 2.5 ± 0.7 MPa in fresh and 2.4 ± 0.5 MPa in thawed specimens, also without a statistically significant difference (mean difference 0.1, 95% CI − 0.5 to 0.7, p = 0.64; Mann-Whitney p = 0.65). Stiffness was 5.2 ± 1.9 N/mm in fresh and 5.4 ± 1.4 N/mm in thawed specimens, with no difference between groups (mean difference − 0.3, 95% CI − 1.8 to 1.3, p = 0.73; Mann-Whitney p = 0.82). Maximum load was 16.0 ± 4.2 N in fresh and 16.7 ± 3.2 N in thawed specimens. The fresh group deviated slightly from normality, but both Welch’s t-test (mean difference − 0.7, 95% CI − 4.2 to 2.8, p = 0.67) and Mann-Whitney *U* (p = 0.82) indicated no significant difference.

Across all four biomechanical parameters, no statistically significant differences were found between fresh and thawed specimens.

#### NCC

**Fig. 5:**
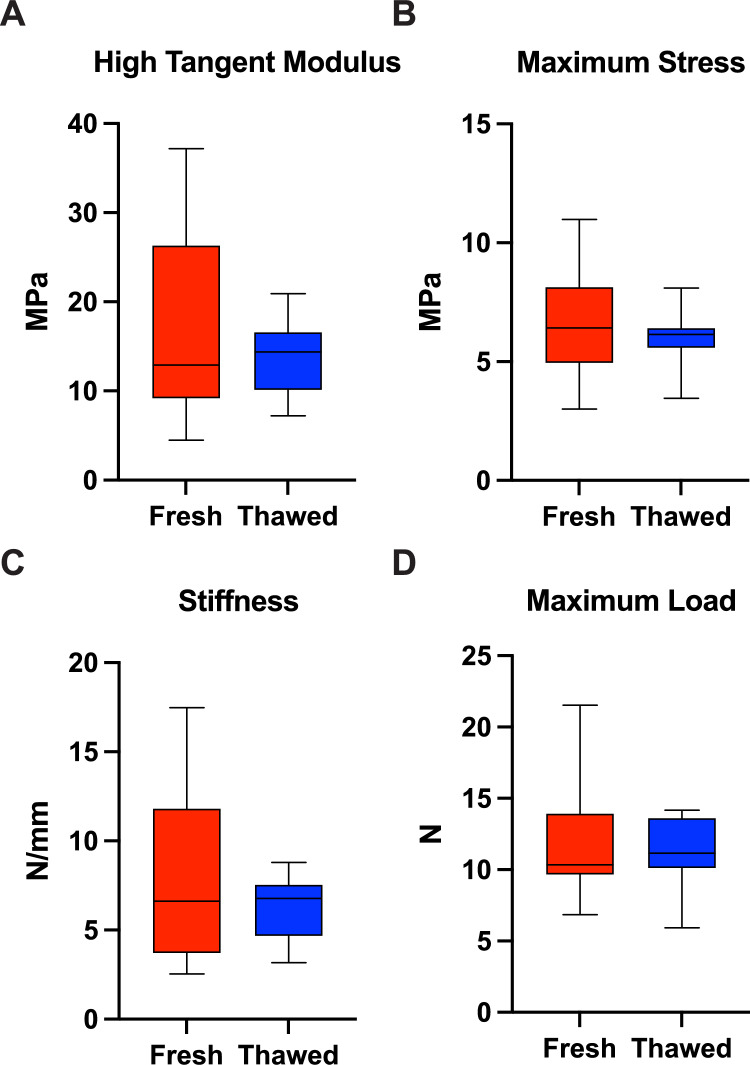
Boxplots illustrating uniaxial tensile test results at the non-coronary cusp in fresh and thawed porcine aortic roots. **A** High-range elastic modulus. **B** Stiffness. **C** Maximum load. **D** Maximum stress. No statistically significant differences were observed between fresh and thawed specimens for any parameter (elastic modulus: p = 0.42; stiffness: p = 0.37; maximum load: p = 0.63; maximum stress: p = 0.47). Boxplots show the median (centre line), interquartile range (box), and whiskers extending to 1.5 × IQR. p-values are from Welch’s t-test; Mann–Whitney *U* was performed as a non-parametric sensitivity analysis

Uniaxial tensile test results for the non-coronary cusp (NCC) are summarized in Table 2 and illustrated in Fig. 5. The high-range elastic modulus was 17.0 ± 11.6 MPa in the fresh group and 13.8 ± 4.1 MPa in the thawed group. Normality was violated in the fresh group (p = 0.047), and both the Welch’s t-test (mean difference 3.2, 95% CI − 5.0 to 11.4, p = 0.42) and the Mann-Whitney U test (p = 0.94) indicated no significant difference. Maximum stress was 6.6 ± 2.5 MPa in fresh and 6.0 ± 1.2 MPa in thawed roots, with no deviation from normality; Welch’s t-test (mean difference 0.6, 95% CI − 1.2 to 2.5, p = 0.47) and Mann-Whitney U (p = 0.55) both showed no difference. Stiffness was 7.7 ± 5.0 N/mm in fresh and 6.2 ± 1.8 in thawed roots; normality was confirmed, and Welch’s t-test (mean difference 1.5, 95% CI − 2.0 to 5.1, p = 0.37) as well as Mann-Whitney U (p = 0.88) indicated no significant difference. Maximum load was 12.0 ± 4.2 N in fresh and 11.3 ± 2.5 N in thawed roots, with normality satisfied; Welch’s t-test (mean difference 0.7, 95% CI − 2.5 to 4.0, p = 0.63) and Mann-Whitney U (p = 0.76) both demonstrated no difference. Across all tested biomechanical parameters, no statistically significant differences were observed between fresh and thawed specimens.

**Table 2:** Tensile test of the NCC, comparing fresh and thawed tissue

Parameter	Fresh (n = 10) mean ± SD	Median (range)	Thawed (n = 10) mean ± SD	Median (range)	Mean difference (95% CI)	Welch’s *t*-test	Mann-Whitney
Tangent modulus, high (MPa)	17.0 ± 11.6	12.9 (4.5–37.2)	13.8 ± 4.1	14.4 (7.2–20.9)	3.2 (− 5.0 to 11.4)	p = 0.42	p = 0.94
Maximum stress (MPa)	6.6 ± 2.5	6.4 (3.0–11.0)	6.0 ± 1.2	6.1 (3.5–8.1)	0.6 (− 1.2 to 2.5)	p = 0.47	p = 0.55
Stiffness (N/mm)	7.7 ± 5.0	6.6 (2.5–17.5)	6.2 ± 1.8	6.8 (3.2–8.8)	1.5 (− 2.0 to 5.1)	p = 0.37	p = 0.88
Maximum load (N)	12.0 ± 4.2	10.4 (6.8–21.5)	11.3 ± 2.5	11.2 (5.9–14.2)	0.7 (− 2.5 to 4.0)	p = 0.63	p = 0.76

Biomechanical parameters of fresh and thawed porcine aortic roots. Values are presented as mean ± standard deviation (SD), median (range), mean differences with 95% confidence intervals (CI), and corresponding p-values from Welch’s two-sample *t*-test and Mann-Whitney *U* test. No statistically significant differences were observed between groups

#### Fluid Dynamics

**Fig. 6:**
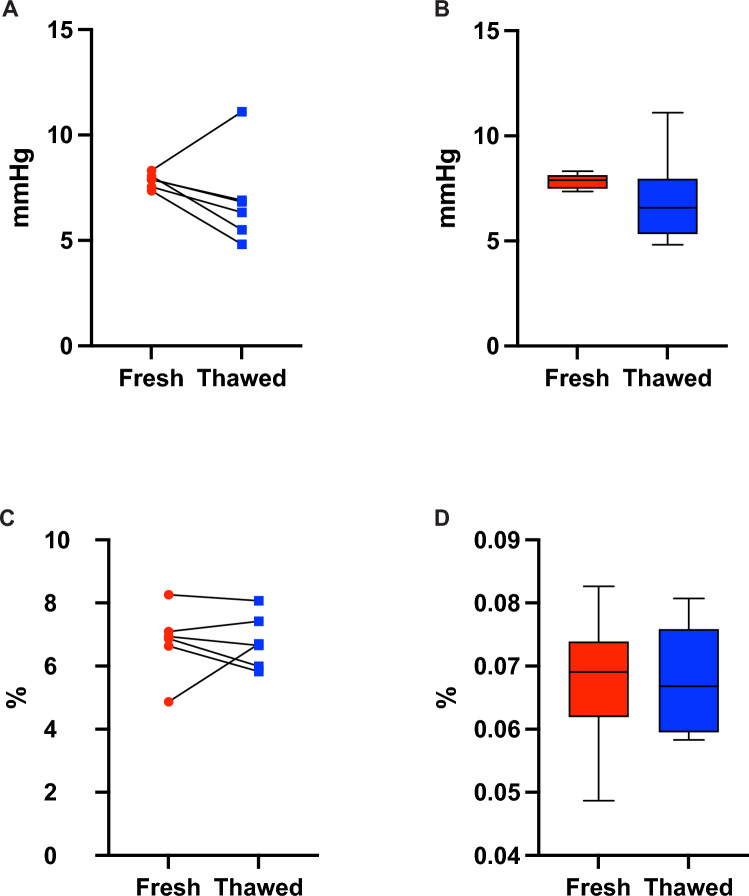
Fluid dynamic performance of fresh and thawed porcine aortic roots. **A** Individual paired measurements of mean aortic root pressure gradient in fresh and thawed specimens. **B** Boxplot summarizing the distribution of mean aortic root pressure gradient across groups. **C** Individual paired measurements of regurgitant fraction in fresh and thawed specimens. **D** Boxplot summarizing the distribution of regurgitant fraction across groups. No statistically significant differences were observed (pressure gradient: p = 0.25; regurgitant fraction: p = 0.997). Boxplots show the median (centre line), interquartile range (box), and whiskers extending to 1.5 × IQR. p-values are from paired *t*-tests

Fluid dynamic data are summarized in Table 3 and depicted in Fig. 6. The mean aortic root pressure gradient was 7.8 ± 0.4 mmHg in the fresh state and 6.8 ± 2.3 mmHg after thawing. The mean paired difference was − 1.1 mmHg (95% CI − 3.2 to 1.0), which was not statistically significant (p = 0.25). In contrast, the regurgitant fraction showed virtually identical values in fresh (6.8 ± 1.1%) and thawed (6.8 ± 0.9%) specimens, with no significant difference detected (mean difference − 0.002 %, 95% CI − 1.1 to 1.1, p = 0.997). In summary, freezing did not significantly alter the aortic root pressure gradient or regurgitant fraction in this model.

**Table 3:** Fluid dynamic data comparing fresh and thawed aortic roots

Parameter	Fresh mean ± SD	Thawed mean ± SD	Mean difference (95% CI)	Paired *t*-test
Mean aortic root pressure gradient (mmHg)	7.8 ± 0.4	6.8 ± 2.3	− 1.1 (− 3.2 to 1.0)	p = 0.25
Regurgitant fraction (%)	6.8 ± 1.1	6.8 ± 0.9	− 0.002 (− 1.01 to 1.1)	p = 0.997

Values are expressed as mean ± standard deviation (SD). Mean difference refers to the paired difference (thawed—fresh). p-value derived from paired *t*-test

#### Ultrasonography

**Fig. 7:**
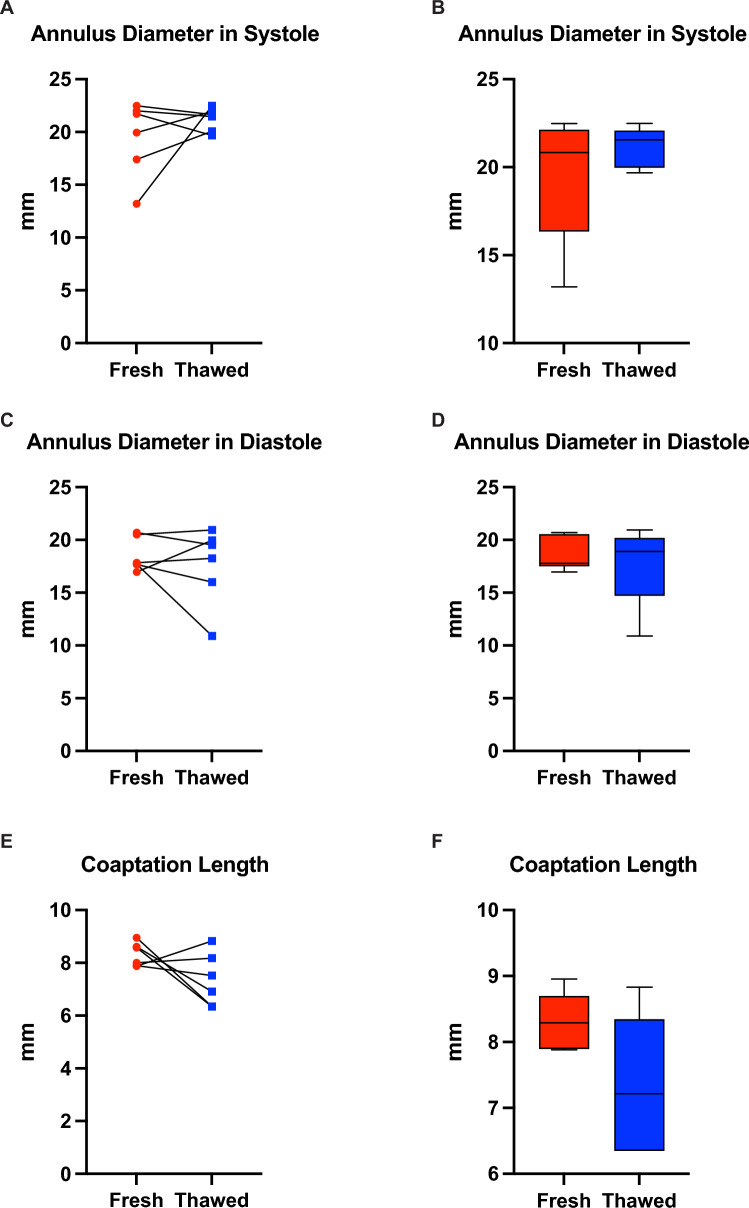
Ultrasonographic measurements in fresh and thawed porcine aortic roots. **A** Individual paired measurements of annular diameter in systole. **B** Boxplot summarizing the distribution of annular diameter in systole. **C** Individual paired measurements of annular diameter in diastole. **D** Boxplot summarizing the distribution of annular diameter in diastole. **E** Individual paired measurements of coaptation length. **F** Boxplot summarizing the distribution of coaptation length. Freeze-thawing did not result in statistically significant changes in any parameter (systolic diameter: p = 0.34; diastolic diameter: p = 0.30; coaptation length: p = 0.14). Boxplots show the median (centre line), interquartile range (box), and whiskers extending to 1.5 × IQR. p-values are from paired *t*-tests

Ultrasonographic measurements are presented in Table 4 and Fig. 7. The mean annular diameter in systole was 19.5 ± 3.6 mm in fresh specimens and 21.2 ± 1.1 mm after thawing. Although there was a tendency towards an increased systolic annular diameter following freezing, the paired comparison did not reach statistical significance (mean difference − 1.8 mm, 95% CI − 6.1 to 2.5, p = 0.34). In diastole, the mean annular diameter was 19.2 ± 2.7 mm in fresh roots compared with 17.6 ± 3.7 mm in thawed roots, corresponding to a non-significant reduction (mean difference − 1.6 mm, 95% CI − 5.1 to 2.0, p = 0.30). Coaptation length was 8.3 ± 0.5 mm in the fresh state and 7.2 ± 1.1 mm after thawing. While coaptation tended to be shorter in thawed specimens, this difference was likewise not statistically significant (mean difference + 1.1 mm, 95% CI − 0.5 to 2.7, p = 0.14). In summary, freeze-thawing did not induce statistically significant changes in annular dimensions or coaptation length; however, consistent trends towards a larger systolic annular diameter and reduced coaptation length were observed in thawed specimens.

**Table 4:** Ultrasonographic data comparing fresh and thawed aortic roots

Parameter	Fresh mean ± SD	Thawed mean ± SD	Mean difference (95% CI)	Paired *t*-test
Annulus diameter, systole (mm)	19.5 ± 3.6	21.2 ± 1.1	− 1.8 (− 6.5 to 2.5)	p = 0.34
Annulus diameter, diastole (mm)	19.2 ± 2.7	17.6 ± 3.7	− 1.6 (− 5.1 to 2.0)	p = 0.30
Coaptation length (mm)	8.3 ± 0.45	7.2 ± 1.1	+ 1.1 (− 0.5 to 2.7)	p = 0.14

Values are expressed as mean ± standard deviation (SD). Mean difference refers to the paired difference (thawed—fresh). *p*-value derived from paired *t*-test

## Discussion

This study investigated whether freezing and subsequent thawing influence the biomechanical and functional characteristics of porcine aortic roots. Across tensile testing, fluid dynamic evaluation, and ultrasonographic imaging, no parameter showed a statistically significant difference. At both the STJ and NCC, tensile properties, including E-module, maximum stress, maximum load, and stiffness, were comparable. Similarly, ultrasonographic measurements of annular geometry and leaflet coaptation showed no statistically significant differences between groups. In the fluid dynamic analysis, the mean aortic pressure gradient, retrograde flow and regurgitant fraction were unaffected. Taken together, these findings indicate that freezing did not alter the structural or functional behavior of porcine aortic roots compared with fresh specimens.

When placed in the context of existing work, our findings align with previous findings on arterial tissue preservation. Stemper et al. reported that frozen arterial specimens maintained tensile strength compared with fresh samples, while refrigerated tissue showed decreased integrity. Refrigerated segments were stored in Ringer’s solution for 24–48 hours at 4 °C and tested within one hour of removal, while frozen specimens were stored for up to 3 months, thawed in a 37 °C water bath, and tested within one hour of thawing [5]. Similarly, O’Leary et al. observed no significant alterations in the biaxial mechanical behavior of porcine descending aortas after long-term freezing [6], and Wilhjelm et al. demonstrated that frozen arteries maintained their geometric configuration on ultrasound [7]. Our findings extend this evidence to the aortic root, a more complex anatomical structure that integrates annular support, cusp motion, and sinotubular dynamics. Importantly, this study provides one of the first systematic evaluations of fresh versus thawed aortic roots, combining tensile testing, in vitro fluid dynamic assessment, and ultrasonography.

Although our findings align with several studies reporting preserved biomechanics after freezing, the literature on vascular tissue preservation is heterogeneous and includes contradictory results. Such discrepancies likely reflect differences in tissue type and anatomical region, storage temperature and duration, freezing protocols, and the mechanical endpoints and testing methods applied.

Accordingly, our conclusions should be interpreted within the conditions tested here: intact porcine aortic roots subjected to a single freeze–thaw cycle at − 20 °C and assessed by uniaxial tensile testing, pulsatile flow-loop analysis, and ultrasonography. Across these macroscopic endpoints, we observed no measurable differences; however, alternative storage conditions, repeated cycles, or other testing modalities may yield different results and warrant further study.

Beyond consistency with prior findings, the absence of measurable differences in tensile properties suggests that extracellular matrix architecture was preserved through a single freeze-thaw cycle at − 20 °C. Structural proteins, such as collagen and elastin, likely retained their integrity, thereby maintaining sample stiffness and load-bearing capacity. This observation supports the use of thawed tissue for biomechanical studies where ultimate strength and elasticity are key outcomes.

Because the aortic root and cusps experience complex multiaxial and cyclic loading in vivo, uniaxial testing primarily informs ultimate tensile behavior and comparative failure-related properties rather than physiological multiaxial compliance and characterization. Freeze–thaw effects, if subtle, may be more apparent under biaxial protocols or cyclic fatigue loading, which were beyond the scope of the present study. Accordingly, these uniaxial metrics should be interpreted as comparative descriptors of high-load mechanical behavior under monotonic loading, rather than as constitutive parameters describing nonlinear, multiaxial, or viscoelastic tissue mechanics.

The lack of group differences in mean aortic pressure gradient, retrograde flow, and regurgitant fraction—parameters that directly reflect valve function—suggests that a single freeze-thaw cycle is unlikely to impair valve competence.

Ultrasonography confirmed these findings, showing similar annular diameters and leaflet coaptation lengths between groups. These imaging data further indicate that freezing and thawing the specimens did not induce geometrical distortion of the aortic root or valve apparatus.

In this study, we used a multimodal approach, integrating biomechanical, fluid dynamic, and imaging assessments, thereby providing a comprehensive evaluation of tissue behavior. The porcine model offers close anatomical and physiological similarity to the human aortic root, enhancing the translational value of the results. Furthermore, the experimental procedures were carefully standardized, including controlled dissection, specimen preparation, a defined freezing-thawing protocol, and predefined statistical analyses. Despite these strengths, several limitations must also be considered when interpreting the results. First, only a single freeze-thaw cycle at a single storage duration (30 days) was evaluated, and the results may not be generalizable to longer storage times or repeated cycles. Second, pure water was used as the operating fluid in the flow-loop, as bulk flow behavior was investigated. However, this may limit the translatability to in vivo haemodynamics. Moreover, the flow-loop protocol was designed to assess acute functional performance and does not evaluate fatigue or cyclic durability; therefore, no conclusions can be drawn regarding long-term functional stability following freeze–thawing. Finally, no histological or molecular analyses were performed, which could have revealed microstructural changes not apparent in functional and macroscopic measurements.

These findings have direct implications for experimental cardiovascular research. The comparable performance of fresh and thawed porcine aortic roots supports the use of freeze-preserved tissue as a practical substitute for fresh specimens. This approach offers practical advantages, including improved standardization across experiments, greater flexibility in scheduling, and reduced dependency on immediate tissue availability from abattoirs. For laboratories employing pulsatile flow-loop models or biomechanical tensile testing, freeze-preserved aortic roots may therefore provide a consistent and accessible resource without compromising biomechanical validity. While these findings are encouraging, additional research is warranted to expand their scope and confirm their applicability in broader experimental contexts.

Future work should address the impact of repeated freeze-thaw cycles and extended storage durations, as such conditions may occur in tissue banking and long-term specimen preservation. Complementary histological and biomechanical analyses are warranted to assess extracellular matrix integrity and identify subtle changes in collagen or elastin organization. Additionally, comparative studies across different freezing protocols and storage temperatures could refine best practices for tissue preservation for experimental cardiovascular research.

In conclusion, freezing and thawing did not measurably alter the biomechanical tensile, fluid dynamic, or geometrical properties of porcine aortic roots under the tested experimental conditions. Within. These findings indicate that thawed tissue can be considered functionally equivalent to fresh tissue in experimental setups, thereby supporting its use as a practical and valid option in cardiovascular research.

## Data Availability

The data used in the current study are available from the corresponding author upon reasonable request.

## Abbreviations

NCC: Non-coronary cusp
STJ: Sinotubular junction
SD: Standard deviation
